# An Analysis of Linker-Dependent Effects on the APC Activation and In Vivo Immunogenicity of an R848-Conjugated Influenza Vaccine

**DOI:** 10.3390/vaccines11071261

**Published:** 2023-07-20

**Authors:** Kali F. Crofts, Courtney L. Page, Stephanie M. Swedik, Beth C. Holbrook, Allison K. Meyers, Xuewei Zhu, Derek Parsonage, Marlena M. Westcott, Martha A. Alexander-Miller

**Affiliations:** 1 Department of Microbiology and Immunology, Wake Forest School of Medicine, Winston-Salem, NC 27101, USA; kcrofts@wakehealth.edu (K.F.C.); cpage@wakehealth.edu (C.L.P.); sswedik@wakehealth.edu (S.M.S.); bcholbro@wakehealth.edu (B.C.H.); am3412@eagleton.rutgers.edu (A.K.M.); xwzhu@wakehealth.edu (X.Z.); mwestcot@wakhealth.edu (M.M.W.); 2 Department of Internal Medicine, Section on Molecular Medicine, Wake Forest School of Medicine, Winston-Salem, NC 27101, USA; 3 Department of Biochemistry, Wake Forest School of Medicine, Winston-Salem, NC 27101, USA; parsonag@wakehealth.edu

**Keywords:** TLR7/8 adjuvants, vaccine, influenza A virus, R848, TLR signaling, dendritic cell

## Abstract

Subunit or inactivated vaccines comprise the majority of vaccines used against viral and bacterial pathogens. However, compared to their live/attenuated counterparts, these vaccines often demonstrate reduced immunogenicity, requiring multiple boosters and or adjuvants to elicit protective immune responses. For this reason, studies of adjuvants and the mechanism through which they can improve inactivated vaccine responses are critical for the development of vaccines with increased efficacy. Studies have shown that the direct conjugation of adjuvant to antigen promotes vaccine immunogenicity, with the advantage of both the adjuvant and antigen targeting the same cell. Using this strategy of direct linkage, we developed an inactivated influenza A (IAV) vaccine that is directly conjugated with the Toll-like receptor 7/8 agonist resiquimod (R848) through a heterobifunctional crosslinker. Previously, we showed that this vaccine resulted in improved protection and viral clearance in newborn nonhuman primates compared to a non-adjuvanted vaccine. We subsequently discovered that the choice of linker used to conjugate R848 to the virus alters the stimulatory activity of the vaccine, promoting increased maturation and proinflammatory cytokine production from DC differentiated in vitro. With this knowledge, we explored how the choice of crosslinker impacts the stimulatory activity of these vaccines. We found that the linker choice alters signaling through the NF-κB pathway in human monocyte-derived dendritic cells (moDCs). Further, we extended our analyses to in vivo differentiated APC present in human peripheral blood, replicating the linker-dependent differences found in in vitro differentiated cells. Finally, we demonstrated in a mouse model that the choice of linker impacts the amount of IAV-specific IgG antibody produced in response to vaccination. These data enhance our understanding of conjugation approaches for improving vaccine immunogenicity.

## 1. Introduction

Inactivated and subunit vaccines are popular vaccine strategies against microbial pathogens as they are generally associated with a strong safety profile. However, inactivated vaccines often demonstrate reduced immunogenicity compared to their live/attenuated counterparts, requiring multiple boosters and or adjuvants to elicit protective immune responses. One solution to this challenge is the use of adjuvants that target innate immune receptors to elicit signals that shape the immune response in a desired way [1,2]. Thus, the study of adjuvants and the mechanism through which they can improve inactivated vaccine responses is essential for developing effective vaccine strategies.

Toll-like receptor (TLR) agonists are a class of adjuvants that have highly immunomodulatory properties resulting in improved vaccine immunogenicity [3,4]. These agonists target a family of pattern recognition receptors (PRRs) called TLRs that are broadly distributed on both innate and adaptive immune cells as well as other non-immune cells. The engagement of TLRs promotes robust cellular activation and maturation following microbial infection [5], which is important in generating appropriate immune responses. Most TLRs are expressed at the cell surface, but a subset (TLR3, TLR7, TLR8, and TLR9) resides within endosomes [6]. This subset of TLRs recognizes nucleic acid structures, i.e., dsRNA (TLR3), ssRNA (TLR7 and TLR8), and unmethylated CpG (TLR9) [2]. During viral infections, ligation of these receptors within antigen-presenting cells (APCs) induces the production of proinflammatory cytokines and upregulates costimulatory molecules that are essential in orchestrating anti-viral immune responses. Our previous work as well as other studies have shown that TLR7/8 agonists are capable of stimulating APC in a manner similar to viral infection, resulting in overall improvements in the immune response [4,7,8,9,10,11,12,13,14,15,16]. Stimulation with TLR7/8 agonists can promote the upregulation of costimulatory molecules (CD40, CD80, and CD86) and TH1 polarizing cytokines in neonatal APC [17,18]. Moreover, a randomized trial of vaccinated elderly individuals who received an inactivated influenza vaccine with topical imiquimod (TLR7 agonist) cream resulted in increased seroconversion compared to individuals who received a vehicle control cream [16]. Thus, there is evidence that supports the utility of TLR7/8 agonists as effective vaccine adjuvants, even in those with immune systems that are less responsive.

It is important to note that the way in which TLR7/8 adjuvants are used in vaccine formulations is critical in generating safe and effective vaccine-elicited responses. Early studies showed that TLR7/8 agonists administered intravenously or orally can result in high reactogenicity [19]. To overcome this, alternative vaccine formulations have been utilized to minimize systemic toxicity by holding the adjuvant at the site of injection. These approaches include lipidation [20,21], encapsulation within nanoparticles [22], adsorption to alum [23], or direct conjugation to protein antigens [9,12,24] or polymers [4,25,26,27,28]. Seder and colleagues have shown that self-aggregating conjugate formulations employing TLR7/8 agonists that form larger particles improve vaccine responses [26,27,29]. A study by Roth et al. has shown that using a crosslinking polymer nanoparticle hydrogel system that contains hemagglutinin and TLR7/8 agonist tethered to the nanoparticle can allow for sustained co-diffusion and delivery of both antigen and adjuvant [28]. In mice, this vaccine showed both an increase in the magnitude and the duration of the antibody response compared to other adjuvants like AddaVax and alum [28]. Overall, TLR7/8-adjuvanted vaccines that use formulations to spatially restrict TLR agonists to the site of injection and draining LNs improve vaccine responses [25]. We have developed an inactivated influenza A virus (IAV) vaccine that directly conjugates a TLR7/8 agonist resiquimod (R848) to influenza virus A/Puerto Rico/8/34 (IPR8) (H1N1) through a heterobifunctional crosslinker [7,8,9,10].

Further supporting our method of direct conjugation are the multiple studies that have demonstrated increased stimulatory capacity of TLR agonists when physically associated with antigen [12,30,31,32]. The advantage of direct linkage lies in targeting both the immunostimulatory component of the adjuvant and the antigen to the same cell, resulting in the optimal maturation of the APC. A study in nonhuman primates (NHPs) showed that vaccination with HIV gag protein directly conjugated to a TLR7/8 agonist increased the quality of the TH1 response and improved the magnitude of antibody produced in comparison to HIV gag protein admixed with the TLR7/8 agonist [12]. Our research has shown that newborn African green monkeys (AGMs) vaccinated with the R848-conjugated IPR8 vaccine have improved influenza-A-specific IFNγ-producing T cell responses, increased antibody production, increased B cell activation, and overall increases in protection when compared to IPR8 with non-adjuvant [8,9]. Thus, the direct linkage of TLR7/8 agonists to antigen results in improved vaccine-elicited immune responses.

In follow-up studies, we screened a series of R848-linker constructs that had similar amide to sulfhydryl bonding chemistry, but varying spacer arm lengths and chemical properties, to determine whether the linker impacted the stimulatory capacity [33]. From this, we identified a candidate vaccine made with the N-γ-maleimidobutyryl-oxysuccinimide ester (GMBS) crosslinker that induced increased proinflammatory cytokine (TNFα, IL-12p70 and IL-6) production and maturation when used to stimulate a murine macrophage cell line and human monocyte-derived dendritic cells (moDCs) compared to the SM(PEG)_4_ crosslinker [33]. The SM(PEG)_4_ crosslinker is what we had previously used in our vaccination studies [7,8,9,10]. The GMBS crosslinker has an aliphatic spacer arm of 7.3 Å as compared to the SM(PEG)_4_ crosslinker which has a hydrophilic spacer arm of 24.6 Å [33].

The goal of the study presented here was to further understand how the choice of a crosslinker impacts the stimulatory activity of these inactivated IAV vaccines and their effect on in vivo differentiated human APC. Additionally, we explored the ability of these vaccines to induce a response in vivo.

## 2. Materials and Methods

*Preparation of vaccine constructs*. The vaccine constructs were prepared using a two-step process that was previously described [9,33]. The first step involved linking a derivative of R848 that was modified to contain a primary amine [9] that allowed for the conjugation to the N-hydroxysuccinimide (NHS) group on a sulfhydryl crosslinker. The two linkers that were used in these studies were SM(PEG)_4_ and GMBS (Thermo Fisher Scientific, Waltham, MA, USA). R848 was incubated with the crosslinkers for 24 h at 37 °C in 5% CO_2_. Conditions were optimized to drive maximum coupling of R848 to the linker. In the second step, R848-linker constructs were incubated with purified IAV strain A/PuertoRico/8/1934 (PR8) (Charles River Avian Vaccine Services, Norwich, CT, USA) for 2 h at 37 °C in 5% CO_2_. The maleimide portion on the linkers allowed for conjugation to the free thiols on PR8. To remove any unbound virus and R848, PR8-linker-R848 constructs were dialyzed for 2 h and then overnight in PBS using 20K Slide-A-Lyzer Mini Dialysis Device (Thermo Fisher Scientific, Waltham, MA, USA). PR8-linker-R848 constructs were then inactivated with 0.74% formaldehyde and incubated for 1 h in a 37 °C water bath before undergoing overnight dialysis. Unconjugated inactivated PR8 (IPR8) was used as a control in these studies (formalin fixed and dialyzed like the other vaccines). The concentration of the vaccine constructs was determined using a Pierce^TM^ BCA protein assay kit (Thermo Fisher Scientific, Waltham, MA, USA) and conjugation was assessed functionally using CD40 upregulation and TNFα production from RAW264.7 stimulated cells [33].

*Differentiation of human moDCs*. Peripheral blood mononuclear cells (PBMCs) were isolated from human blood from male and female donors aged 25–40 years old (ZenBio) using density gradient centrifugation. CD14+ cells were isolated by positive selection using human CD14 MicroBeads (Miltenyi Biotech). Human moDCs were differentiated using 10^7^ CD14+ cells (10^6^ cells/mL) cultured in 10 mL of RPMI-1640 medium supplemented with 2 mM L-glutamine, 100 U/mL penicillin, 100 µg/mL streptomycin (Gibco), 0.05 mM β-ME (Sigma Aldrich, St. Louis, MO, USA), 10% FBS (Atlanta Biologicals, Lawrenceville, GA, USA), 10 ng/mL human GMCSF, and 10 ng/mL of human IL-4 for 6 days at 37 °C in 5% CO_2_ [34]. On day 3, half of the medium was replaced with fresh cytokine-supplemented media. On day 6, cultures were more than 95% CD11c+ [33].

*Vaccine-stimulated moDCs for kinetic assessment of intracellular cytokine production*. Day 6 human moDCs were plated (2.5 × 10^5^ cells/well) overnight in 48-well plates (RPMI, 10% FBS (Atlanta Biologicals, Lawrenceville, GA, USA), 2 mM L-glutamine, 100 U/mL penicillin, 100 µg/mL streptomycin (Gibco, Billings, MT, USA), 0.05 mM β-ME (Sigma Aldrich, St. Louis, MO, USA), 10 ng/mL human GMCSF, and 10 ng/mL of human IL-4). Human moDCs were stimulated in vitro for 12 or 24 h with 10 µg/mL of IPR8, IPR8-SM(PEG)_4_-R848, IPR8-GMBS-R848, 10 µM R848 (Enzo Life Sciences, Farmingdale, New York, NY, USA) alone to serve as a positive control, or were left non-stimulated (NS). IPR8 was originally titrated to a level that does not induce a strong proinflammatory response so that the effects of the adjuvanted vaccine constructs could be optimally evaluated. For the dose titration of R848, cells were stimulated with either 10, 25, or 60 µM of R848. Intracellular cytokine production from immune populations were measured between 12 and 24 h in the presence of Brefeldin A (1:1000, GolgiPlug, BD Biosciences, Franklin Lakes, NJ, USA) and Monensin (1:1500, GolgiStop, BD Biosciences, Franklin Lakes, NJ, USA). Cells were pelleted and stained with Zombie Aqua fixable viability kit (Biolegend, San Diego, CA, USA) followed by surface staining with anti-CD11c APC (S-HCL-3) (Biolegend, San Diego, CA, USA). Cells were fixed and permeabilized using BD Cytofix/Cytoperm Plus kit (BD Biosciences, Franklin Lakes, NJ, USA) followed by staining with anti-TNFα AF488 (CD25-4E3) (BD Biosciences, Franklin Lakes, NJ, USA) and anti-IL-12p70 PE (20C2) (BD Biosciences, Franklin Lakes, NJ, USA). Samples were acquired on a BD LSRFortessa X-20 (BD Biosciences, Franklin Lakes, NJ, USA) and analyzed with BD FACSDiva Software (BD Biosciences, Franklin Lakes, NJ, USA).

*Determining the amount of R848 conjugated to the inactivated PR8 vaccines using spectrophotometry*. The extinction coefficient (4581 M^−1^cm^−1^) was determined for 10 µM of R848 at 316 nm using the equation, extinction coefficient for R848 = Peak absorbance of R848 (at 316 nm)/concentration of R848 (10 µM). We then measured the absorbance of each vaccine on the spectrophotometer (Varian, Palo Alto, CA, USA) at 100 µg/mL using the Varian Software version 3.00. We corrected the vaccine constructs to IPR8 alone and then determined the change in absorbance between the vaccine constructs (IPR8-SM(PEG)_4_ and IPR8-GMBS-R848) to IPR8 at 317 nm and 320 nm, respectively (this is where we observed the peak R848 absorbance for each vaccine construct). The change in absorbance was then divided by the extinction coefficient for R848.

*Dynamic light scattering to assess the aggregation of the vaccine constructs***.** Vaccine samples were prepared at 100 µg/mL and centrifuged at 5900× *g* for 5 min to remove any dust. Samples (100 µL) were placed in a cuvette and run on the ZetaSizer Nano Z6 (Malvern Panalytical, Worcestershire, UK) equipped with a 633 nM laser. A total of 10 measurements were made for each sample run at 25 °C ± 0.1 °C. The cumulative Z-Average and polydispersity index (PDI) was automatically determined using the ZetaSizer software.

*Assessment of IL-8 production from vaccine stimulated human TLR7 HEK cells*. 293XL-hTLR7 cells (InvivoGen, San Diego, CA, USA) (1 × 10^5^ cells/well) were cultured in 96-flat bottom plates overnight. Cells were cultured in DMEM, 4.5 g/L glucose, 10% FBS (Atlanta Biologicals, Lawrenceville, GA, USA), 50 U/mL penicillin, 50 µg/mL streptomycin, 2 mM L-glutamine (Gibco, Billings, MT, USA), and 10 µg/mL of Blasticidin (InvivoGen, San Diego, CA, USA) (selective antibiotic). As a negative control, HEK-Blue Null1 cells (InvivoGen, San Diego, CA, USA) (4 × 10^4^–1 × 10^5^ cells/well) were cultured in 96-flat-bottom plates. Cells were stimulated in vitro for 24 h with a dose titration (20 µg/mL–1.25 µg/mL) of IPR8, IPR8-SM(PEG)_4_-R848, or IPR8-GMBS-R848. Cells were also stimulated with a dose titration (10 µM–0.078 µM) of R848 (Enzo Life Sciences, Farmingdale, New York, NY, USA) alone to serve as a positive control, or were left non-stimulated (NS). Supernatant was harvested after 24 h and the IL-8 production was measured using a human IL-8 ELISA Max kit (Biolegend, San Diego, CA, USA). The concentration of IL-8 was calculated per the manufacturer’s instructions using the kit’s standard. We determined the relative IL-8 production from the cells by normalizing the concentration of IL-8 at each vaccine dose relative to the maximum concentration of IL-8 made in the positive control R848 stimulated cells (5–10 µM) for each experiment. This normalization approach was used to allow for the analysis of the IL-8 response to each vaccine in a manner that accounts for any potential variations in cell line responsiveness across independent experiments.

*SDS-PAGE and immunoblot analysis*. On day 6 of culture, human moDCs cells were plated overnight in 48-well plates and stimulated on day 7 for 45, 90, 135, or 180 min. Cells were lysed with radioimmunoprecipitation (RIPA) buffer containing a protease inhibitor cocktail, and protein concentration was assessed using a Pierce^TM^ BCA protein assay kit (Thermo Fisher Scientific, Waltham, MA, USA). Protein samples (12.5 µg) were separated using 10% polyacrylamide gel electrophoresis, followed by a transfer onto an ImmunoBlot PVDF membrane (Bio-Rad, Hercules, CA, USA). Membranes were blocked in 5% dried milk in TBST (20 mM Tris, 150 mM NaCl, 0.1% Tween-20, pH 7.6) and incubated overnight at 4 °C with either: mouse anti-phospho IκBα (H.709.9) (1:500 dilution) (Thermo Fisher Scientific, Waltham, MA, USA), mouse anti-IκBα (6A920) (1:1000 dilution) (Thermo Fisher Scientific, Waltham, MA, USA), or mouse anti-GAPDH (6C5) (1:3000) (Santa Cruz Biotechnology, Dallas, TX, USA). Membranes were washed 3 × 10 min with TBST, then treated with secondary antibody goat anti-mouse IgG (H + L) conjugated to HRP (1:10,000 dilution) (Thermo Fisher Scientific, Waltham, MA, USA) for 1 h at RT. Membranes were washed 2× with TBST for 10 min, followed by 2 × 10 min washes with TBS (20 mM Tris, 150 mM NaCl, pH 7.6). Protein was detected using Pierce^TM^ ECL Western Blotting Substrate (Thermo Fisher Scientific, Waltham, MA, USA). For additional antibody detection, membranes were washed 3× with a mild stripping buffer (15 g glycine, 1 g SDS, 10 mL Tween-20, pH 2.2, q.s. to 1 L with H_2_O) for 5 min, washed 2× with PBS (137 mM NaCl, 2.7 mM KCl, 8.1 mM Na_2_HPO_4_, 1.5 mM KH_2_PO_4_) for 10 min, and 2× TBST for 5 min. Following stripping, membranes were blocked in 5% milk in TBST before adding additional primary detection antibodies. ImageJ was utilized to quantify immunoblot protein band pixel density. To do this, a region surrounding the band of interest was selected, and pixel density was measured. To account for background signal, an identically sized region adjacent to the band of interest was measured and this value was subtracted from the protein band density measured. This process was repeated for all protein bands measured. The ratios reported were the ((phospho-IκBα Band—Background)/(Total IκBα Band—Background)) and ((Total IκBα Band—Background)/(GAPDH Band—Background)).

*Vaccine-stimulated PBMCs for assessment of intracellular cytokine production*. PBMC cultures (1 − 2 × 10^6^ cells/well) were maintained in 48-well plates in RPMI-1640 medium supplemented with 2 mM L-glutamine, 1 mM sodium pyruvate, 1× non-essential amino acids, 100 U/mL penicillin, 100 µg/mL streptomycin, 10 mM HEPES (Gibco, Billings, MT, USA), 0.05 mM β-ME (Sigma Aldrich, St. Louis, MO, USA), and 10% FBS (Atlanta Biologicals, Lawrenceville, GA, USA). PBMCs were stimulated in vitro for 16 h with 1 µg/mL or 10 µg/mL of IPR8, IPR8-SM(PEG)_4_-R848, IPR8-GMBS-R848, or a TLR cocktail consisting of 1 µg/mL MPLA (InvivoGen, San Diego, CA, USA), 1 µg/mL PolyI:C (InvivoGen, San Diego, CA, USA), 10 µM R848 (Enzo Life Sciences, Farmingdale, New York, NY, USA) to serve as a positive control, or were left non-stimulated (NS). Following stimulation, surface analysis of immune populations was performed. Cells were pelleted and stained with Zombie Aqua fixable viability kit (Biolegend, San Diego, CA, USA) followed by surface staining with anti-CD3 PE-Cy5 (HIT3a) (BD Biosciences, Franklin Lakes, CA, USA), anti-CD20 BUV395 (2H7) (BD Biosciences, Franklin Lakes, CA, USA), anti-HLA-DR PE-CF594 (G46-6) (BD Biosciences, Franklin Lakes, CA, USA), anti-CD14 PerCpCy5.5 (HCD14) (BioLegend, San Diego, CA, USA), and anti-CD11c APC (S-HCL-3) (Biolegend, San Diego, CA, USA). Intracellular cytokine production from immune populations was measured between 4 and 16 h in the presence of Brefeldin A (1:1000, GolgiPlug, BD Biosciences, Franklin Lakes, CA, USA) and quantified using BD Cytofix/Cytoperm Plus kit (BD Biosciences, Franklin Lakes, CA, USA) followed by staining with anti-TNFα AF488 (CD25-4E3) (BD Biosciences, Franklin Lakes, CA, USA). Samples were acquired on a BD LSRFortessa X-20 (BD Biosciences, Franklin Lakes, CA, USA) and analyzed with BD FACSDiva Software (BD Biosciences, Franklin Lakes, CA, USA).

*TNFα and IL-12p70 ELISA*. Supernatant was harvested from human PBMCs at 24 h following stimulation with 1 µg/mL or 10 µg/mL of IPR8, IPR8-SM(PEG)_4_-R848, IPR8-GMBS-R848, or were left non-stimulated (NS). TNFα production was measured using the human TNFα ELISA Max kit (Biolegend, San Diego, CA, USA). IL-12p70 production was measured using the human IL-12p70 ELISA Max kit (Biolegend, San Diego, CA, USA). IFNα production was measured using the human IFNα2 ELISA Max Deluxe kit (Biolegend, San Diego, CA, USA). The concentration of each cytokine was calculated per the manufacturer’s instructions using the kit’s standard.

*Mouse Vaccination*. Three-week old BALB/c mice were vaccinated intramuscularly (i.m) with 0.03 µg of IPR8, IPR8-SM(PEG)_4_-R848 or IPR8-GMBS-R848. Serum was isolated from mice 14 days post vaccination (p.v.) and the PR8-specific IgG antibody was assessed. Naïve 6-week old BALB/c mouse serum was used as a non-vaccinated negative control that approximated the age of the vaccinated animals following 14 days p.v.

*PR8-specific IgG ELISA*. To measure PR8-specific IgG antibody, half-area microplates (Griener, Monroe, LA, USA) were coated with 0.35 µg of purified A/PuertoRico/8/1934 (PR8) (Charles River Avian Vaccine Services, Norwich, CT, USA) in coating buffer (8.4 g NaHCO_3_, 3.56 g Na_2_CO_3_, add deionized water to 1.0 L, pH to 9.5, 0.2 mM filtered. The final molarity of NaHCO_3_ = 0.1 M, and Na_2_CO_3_ = 0.034 M) overnight at 4 °C. Plates were blocked with 1× blocking buffer (10× casein blocking buffer) (Sigma-Aldrich, St. Louis, MO, USA) for 1 h and then washed. Serum samples were serially diluted in 1× blocking buffer. Wells that contained no virus served as a negative control. Horseradish peroxidase (HRP) conjugated antibody specific for mouse IgG (NA931V) (Amersham, GE Healthcare, Chicargo, IL, USA), was used to detect bound PR8-specific antibodies. Plates were developed using 3, 3′, 5, 5′—tetra methylbenzidine dihydrochloride (TMB) (Sigma-Aldrich, St. Louis, MO, USA) and read at 450 nm on an Elx800 absorbance microplate reader (BioTek, Winooski, VT, USA). For each dilution, the OD from the non-virus-coated wells was subtracted from the virus-coated wells. The assay background was determined by averaging PR8-coated wells that only received the 1× blocking buffer. The threshold titer (TT) was defined at the dilution that reached an absorbance above 3× the assay background.

*Statistical Analysis*. Statistical analyses were performed using Prism 9.2.0 (GraphPad, La Jolla, CA, USA) and the results are represented as the mean or ± SEM. Post hoc Tukey’s or Fisher’s LSD multiple comparison analyses were performed. *p* values were defined as * *p* = < 0.05, ** *p* = < 0.005, *** *p* = < 0.001, **** *p* = < 0.0001).

## 3. Results

### 3.1. The GMBS-Containing Vaccine Promotes Proinflammatory Cytokine Production to a Higher Level and in an Increased Percentage of Human moDCs Compared to the SM(PEG)_4_-Containing Vaccine

We have developed a strategy for conjugating the TLR7/8 agonist R848 to IAV through a heterobifunctional crosslinker [7,8,9,10]. The R848 utilized is an amine derivative that binds to the reactive NHS group of the heterobifunctional crosslinker, allowing the maleimide group to bind to the free thiols that are available on the surface of the PR8 virion. Previously, we have shown that the choice of crosslinker impacts the capacity of the R848-vaccine conjugate to stimulate moDC maturation and proinflammatory cytokine production [33]. Here, we extended our analysis to determine whether the increased cytokine observed at the population level was due to increased production on a per cell basis, an increase in the number of cells producing cytokine, or both.

**Figure 1:**
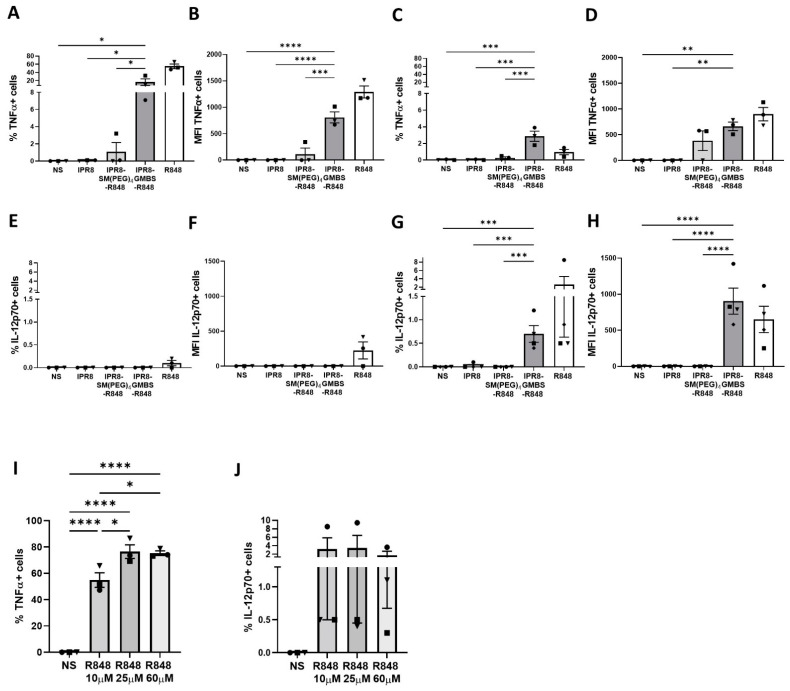
The choice of linker impacts the percentage of human moDCs responding to R848-conjugated influenza virus vaccines as well as the amount of cytokine produced. CD14+ PBMCs were isolated from healthy adult donors and cultured for 7 days in the presence of 10 ng/mL of human IL-4 and GM-CSF. Human moDCs were stimulated in vitro with IPR8, IPR8-SM(PEG)_4_-R848, IPR8-GMBS-R848 (all vaccines 10 μg/mL), R848 (10 µM), or non-stimulated (NS). Intracellular cytokine production was determined using flow cytometry. The percentage of TNFα + CD11c+ cells and the MFI were assessed between 0 and 12 h (**A**,**B**) and 12 and 24 h (**C**,**D**) post stimulation, respectively. The percentage of IL−12p70 + CD11c+ cells and the MFI were assessed between 0 and 12 h (**E**,**F**) and 12 and 24 h (**G**,**H**) post stimulation, respectively. Human moDCs were also stimulated with different concentrations of soluble R848 (10 µM, 25 µM, and 60 µM) and the percentage and MFI of TNFα + CD11c+ cells (**I**) and IL−12p70 + CD11c+ (**J**) were assessed. The data represent mean ± SEM from 3–4 donors. Symbols on graph represent individual donors. Statistical significance for all vaccine stimulations (excluding R848) was assessed using a one-way ANOVA with post hoc Fisher’s LSD multiple comparison analysis. * *p* = < 0.05, ** *p* = < 0.005, *** *p* = < 0.0005, **** *p* = < 0.0001.

We isolated CD14+ PBMCs from healthy donors and differentiated them into moDCs. Cytokine production was measured via intracellular cytokine staining (ICS) at 12 h and 24 h following stimulation with IPR8, IPR8-SM(PEG)_4_-R848, IPR8-GMBS-R848, soluble R848 (positive control), or non-stimulated (NS). The percentage of TNFα+ cells was higher in cells stimulated with IPR8-GMBS-R848 at both time points compared to IPR8-SM(PEG)_4_-R848 stimulated cells (Figure 1A,C). The higher percentage of cells observed 12 h post stimulation (Figure 1A) was associated with higher TNFα expression on a per-cell basis compared to IPR8-SM(PEG)_4_-R848 stimulated cells within the producing population (Figure 1B). Next, we evaluated the percentage of IL-12p70-producing cells in vaccine-stimulated moDCs. We observed a relatively small subset of cells producing IL-12p70 (Figure 1E,G). As with TNFα, IPR8-GMBS-R848 promoted an increase in the percentage of IL-12p70+ cells (Figure 1G) and increased IL-12p70 on a per-cell basis compared to the IPR8-SM(PEG)_4_-R848 stimulated cells, in which no IL-12p70 was detected (Figure 1H). In contrast to TNFα, the greatest response was present at 24 h following stimulation (Figure 1G,H) compared to cells stimulated at 12 h (Figure 1E,F).

The finding that a much smaller percentage of cells produced IL-12p70 versus TNFα led us to question whether this reflected a limited subset of moDCs with the capability to produce this cytokine or whether the stimulation was inadequate to induce production. To address this, we stimulated moDCs with a range of R848 concentrations to determine whether increasing the stimulation would result in an increase in the percentage of cells making cytokine. We chose R848 as a stimulator that could be added at high concentrations to optimally test this question. Our data showed that increasing the concentration of R848 from 10 µM to 25 µM resulted in an increase in the percent of TNFα+ CD11c+ cells (Figure 1I). Increasing the R848 to 60 µM did not result in further increases (Figure 1I), showing that we had reached the maximal stimulatory activity for R848. In contrast, we did not observe a dose-dependent increase in the IL-12p70+ cells (Figure 1J) following addition of 25 or 60 µM R848. These results indicate that only a small subset of moDCs have the capacity to produce IL-12p70 in response to R848. Together, these data show that the GMBS-linked vaccine promotes increases in both the percentage of cells capable of producing proinflammatory cytokine and the expression of cytokine on a per-cell basis compared to the SM(PEG)_4_-linked vaccine.

### 3.2. GMBS and SM(PEG)_4_ Vaccine Constructs Have Similar Amounts of R848 Conjugated to the Inactivated Virion

We next wanted to explore how these different linker constructs might contribute to the changes observed in stimulatory capacity. Firstly, we sought to gain insights into the hypothesis that the increased stimulatory activity was a result of increased R848 conjugation to the virus. To test this, we determined the amount of R848 associated with each vaccine using spectrophotometry. The extinction coefficient was determined for R848 (316 nm), which was used to quantify the concentration of R848 per 100 µg of each vaccine. The amount of R848 on each vaccine was similar, 8.12 nmol of R848 per 100 µg of IPR8-SM(PEG)_4_-R848/mL and 6.05 nmol of R848 per 100 µg of IPR8-GMBS-R848/mL (Table 1). This direct measurement of R848 is in agreement with our previous data that used the loss of free thiols on the virion following conjugation with the virus, a method limited by the indirect nature of measuring R848 [33]. Together with our previous studies, these data strongly support the conclusion that the improved stimulatory activity observed with the GMBS vaccine construct is not a result of an increased amount of R848 on the virion.

### 3.3. Differential Aggregation Does Not Account for the Stimulatory Activity Observed with the Vaccine Constructs

**Figure 2:**
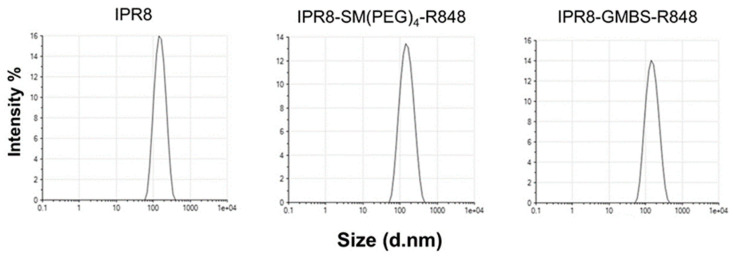
The choice of linker does not alter the aggregation of the vaccines. Dynamic light scattering was performed on IPR8, IPR8-SM(PEG)_4_-R848, and IPR8-GMBS-R848 to assess uniformity.

Protein aggregates found within vaccines have been shown to promote increases in vaccine immunogenicity [35,36]. Therefore, we evaluated whether vaccines produced with the SM(PEG)_4_ versus GMBS linkage differed in their ability to aggregate. We used dynamic light scattering (DLS) for quantitative analysis of the size and uniformity of the particles within each vaccine. Particle size distribution and polydispersity index (PDI) were measured for each vaccine (Figure 2). The Z-average is an intensity weighted mean hydrodynamic size of a collection of particles measured using DLS. We observed similar Z-averages when we compared IPR8-SM(PEG)_4_-R848 (Z-average = 138.3 d.nm) to IPR8-GMBS-R848 (Z-average = 138.6 d.nm) (Table 1). Each vaccine displayed a single peak, the intensities of which were similar between the two adjuvanted vaccines (Figure 2). PDI is used as a measure of the heterogeneity, i.e., aggregation within samples. We observed that for both of the conjugated vaccines, the PDI increased slightly compared to the IPR8 (Figure 2); however, the PDI (IPR8-SM(PEG)_4_-R848, PDI = 0.137 and IPR8-GMBS-R848, PDI = 0.129) of the two conjugated vaccines was similar. Thus, these data show that the choice of linker does not impact the uniformity of the adjuvanted vaccines, suggesting that the increased stimulatory capacity observed with the GMBS vaccine is not a result of altered aggregation.

### 3.4. The Linker-Dependent Differences in Stimulatory Activity Are Dependent on TLR7/8 Engagement

**Figure 3:**
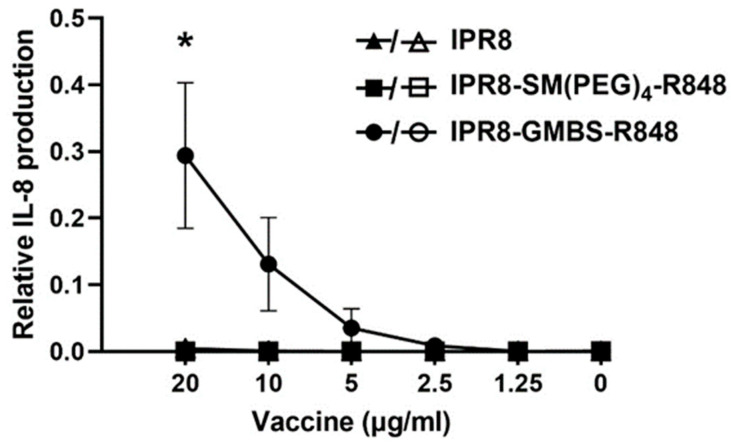
Stimulation with IPR8-GMBS-R848 results in IL-8 production from human TLR7 HEK cells. HEK XL-293 hTLR7 cells were stimulated in vitro with IPR8, IPR8-SM(PEG)_4_-R848, IPR8-GMBS-R848 (20 μg/Ml–1.25 μg/mL) (closed symbols), and R848 (10 µM–0.078 µM), or were left non-stimulated (NS). Supernatants were harvested 24 h following stimulation and IL-8 production was measured using ELISA. To assess the potential for TLR7 independent IL-8 production, HEK-Blue Null1 cells were similarly stimulated (open symbols). The relative amount of IL-8 production was determined by normalizing the concentration of IL-8 (pg/mL) at each vaccine dose relative to the maximum concentration of IL-8 (pg/mL) made in the positive control R848 stimulated cells for each experiment. The data represent mean ± SEM from n = 3 (hTLR7 HEK cells) and n = 2 (Null1 cells). Each experimental repeat (n) was performed independently on different days and from different cell passages. A one-way ANOVA with post hoc Tukey’s multiple comparison analysis was performed. Statistical significance was determined in cells stimulated with 20 µg/mL IPR8-GMBS-R848 when compared against cells stimulated with IPR8 or IPR8-SM(PEG)_4_-R848 (* *p* = < 0.05).

We have previously shown that the choice of the linker can alter the stimulatory activity of murine RAW264.7 macrophages and human moDCs [33]. These findings are consistent with the ability of the R848-conjugated vaccines to activate through TLR7 (RAW264.7) [37] as well as TLR8 (moDCs) [33,38,39]. While the most likely possibility to explain the altered responses of the two vaccines was through binding TLR7/8, the presence of the linkers might impact how the vaccines engaged with other pathways. If this were the case, we reasoned that the differential stimulatory capacity of the GMBS- versus SM(PEG)_4_-containing vaccine might be independent of TLR7/8. To formally test this, we utilized a human TLR7-transfected 293XL HEK cell line, which allowed for the assessment of TLR7-dependent stimulatory activity as a readout of IL-8 production together with its null counterpart, Null1. HEK293 cells express endogenous TLR1, TLR3, TLR5, TLR6, and NOD. TLR7 expressing HEK293 cells or Null1 cells were stimulated with titrated amounts of IPR8, IPR8-SM(PEG)_4_-R848, and IPR8-GMBS-R848 or with R848 for 24 h. IL-8 production was determined for each vaccine construct relative to R848 (Figure 3). Cells stimulated with IPR8-GMBS-R848 had a dose-dependent production of IL-8. IL-8 was undetected in cells stimulated with IPR8-SM(PEG)_4_-R848 or IPR8. IL-8 was undetected in the Null1 cells, regardless of the vaccine used, supporting the conclusion that IL-8 production is TLR7 dependent. We appreciate that this study does not rule out all potential TLR7/8-independent activity. Still, the increased stimulatory activity of IPR8-GMBS-R848 in the TLR7-expressing cells along with the lack of stimulation in the Null1 cells strongly supports the hypothesis that the increased stimulatory activity of the IPR8-GMBS-R848 vaccine is mediated through its engagement with TLR7/8.

### 3.5. IPR8-GMBS-R848 Promotes Increases in IκBα Phosphorylation Compared to IPR8-SM(PEG)_4_-R848

We hypothesized that the differences observed in the stimulatory activity of these vaccines resulted from differential TLR signaling. As human moDCs preferentially respond to TLR8 agonists [33,38,39], we expect the vaccines to signal through TLR8/MyD88 in these cells. Signaling through TLR8 leads to the activation of multiple transcription factors including Nuclear Factor kappa B (NF-κB), subsequently producing proinflammatory cytokines. Therefore, we assessed whether the differences in cytokine production observed with the vaccine constructs were associated with differential NF-κB activation. In the resting state, NF-κB is sequestered in the cytoplasm via the inhibitor of NF-κB (IκB) family members that mask the signal important for nuclear translocation [40]. Upon cell stimulation, IκBα is phosphorylated by IκB kinase (IKK), which results in the ubiquitin-mediated degradation of IκBα, allowing for the nuclear translocation of NF-κB subunits and the transcription of proinflammatory cytokine genes [41].

**Figure 4:**
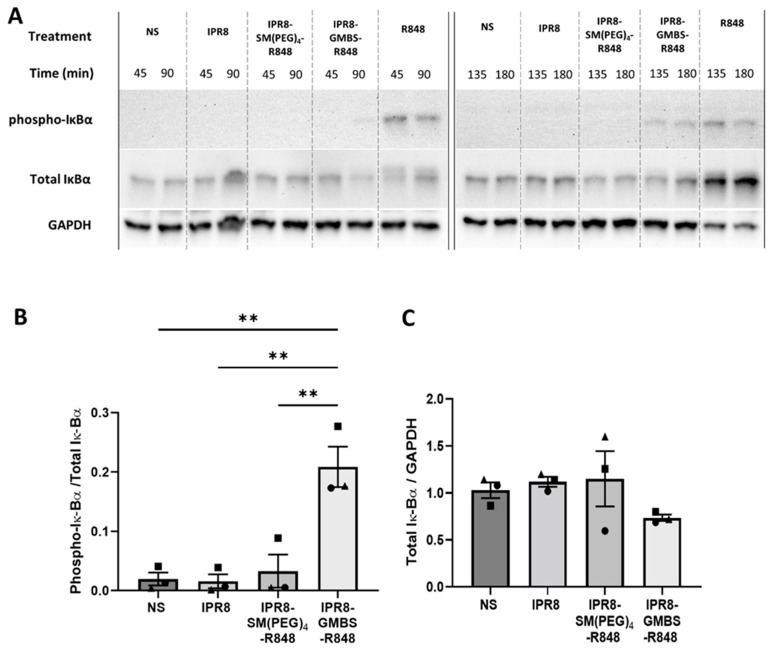
Stimulation with IPR8-GMBS-R848 leads to increased phosphorylation of IκBα compared to IPR8-SM(PEG)_4_-R848. CD14+ PBMCs were isolated from healthy adult donors and cultured for 7 days in the presence of 10 ng/mL of human IL-4 and GM-CSF. Resulting moDCs were stimulated in vitro with 10 μg/mL IPR8, IPR8-SM(PEG)_4_-R848, or IPR8-GMBS-R848, R848 alone (10 µM), or non-stimulated (NS) for 45, 90, 135, or 180 min. Cell lysates (12.5 μg) were run on a 10% SDS-PAGE gel. Phospho- IκBα and total IκBα were detected via immunoblot. GAPDH was used as a loading control. (**A**) Blot is representative of three independent experiments. Immunoblot results from the 135 min stimulation were quantified and reported as ratios of phospho-IκBα/Total IκBα (**B**) or Total IκBα/GAPDH (**C**). Statistical analysis was completed using a one way ANOVA with post hoc Tukey’s multiple comparison analysis. The data represent mean ± SEM from 3 donors. Symbols on graph represent individual donors. ** *p* = < 0.005.

To assess NF-κB activity, we measured the phosphorylation of IκBα and the total IκBα in vaccine-stimulated human moDCs over time (45, 90, 135, and 180 min) (Figure 4A). Soluble R848 was used as a positive control for the activation of the NF-κB pathway (Figure 4A). The ratio of phospho-IκBα/total IκBα was calculated at 135 min after vaccine stimulation to determine the differences in NF-κB activation between the different vaccines. This time point was selected based on the differences in IκBα phosphorylation seen on the immunoblots. We observed a statistical increase in the phospho-IκBα/total IκBα ratio in cells stimulated with the IPR8-GMBS-R848 vaccine compared to the IPR8-SM(PEG)_4_-R848 and the IPR8 vaccines (Figure 4B). To ensure the reported differences in IκBα phosphorylation were not driven by changes in total IκBα expression, we also calculated the ratio of total IκBα/GAPDH (Figure 4C). The comparable levels of total IκBα in the different vaccine stimulated groups supports the finding that there is increased activation of NF-κB in the IPR8-GMBS-R848 stimulated moDCs. The modest decrease in the total IκBα signal in these cells may suggest degradation of this protein (Figure 4C), which would, in turn, further activate NF-κB. Taken together, these data suggest increased NF-κB activation leads to the increased proinflammatory cytokine production observed in IPR8-GMBS-R848-stimulated moDCs (Figure 1 and [33]).

### 3.6. The GMBS Vaccine Construct Promotes Proinflammatory Cytokine Production from APC within the PBMCs

**Figure 5:**
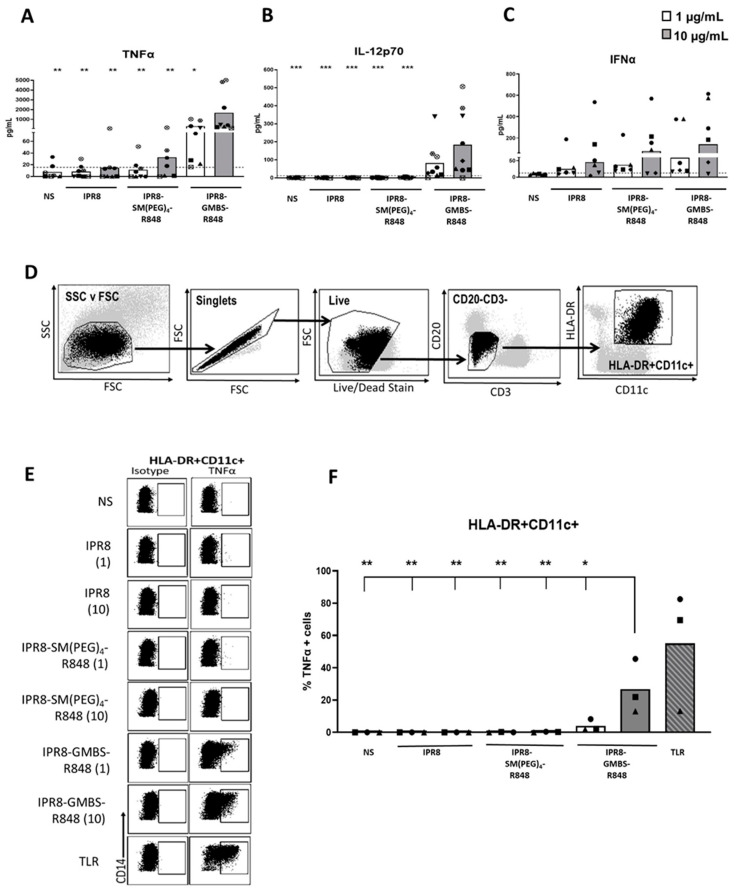
Antigen-presenting cells within PBMCs produce significantly more TNFα when stimulated with IPR8-GMBS-R848 compared to stimulation with IPR8-SM(PEG)_4_ or IPR8 alone. PBMCs were isolated from healthy adult donors and stimulated ex vivo with IPR8, IPR8-SM(PEG)_4_-R848, IPR8-GMBS-R848 (1 μg/mL and 10 μg/mL), or were left non-stimulated (NS). Supernatants were harvested 24 h following stimulation and (**A**) TNFα, (**B**) IL-12p70, and (**C**) IFNα production was measured using ELISA. For flow cytometry analysis, PBMCs were isolated from healthy adult donors and stimulated ex vivo with IPR8, IPR8-SM(PEG)_4_-R848, IPR8-GMBS-R848 (all vaccines 1 μg/mL and 10μg/mL), a TLR cocktail as a positive control (MPLA 1 μg/mL, PolyI:C 1 μg/mL, and R848 10 μM), or were left non-stimulated (NS) for 16 h. Brefeldin A was added to the cell cultures for a maximum of 12 h (present during 4–16 h of stimulation) to perform ICCS. After stimulation, cells were collected and stained for marker expression and cytokine production. (**D**) APCs were identified as live cells that are CD3- CD20- HLA-DR+ CD11c+. (**E**) Representative flow plots of TNFα producing APCs (HLA-DR+ CD11c+). (**F**) The percentage of TNFα+ cells (HLA-DR+ CD11c+). Data represent mean from 6–9 donors for the ELISA and 3 donors for flow cytometric analysis. Symbols on graph represent individual donors. Statistical significance for all vaccine stimulations (not including TLR mix) was assessed using one-way ANOVA to compare the mean of each vaccine stimulation with the mean of all other vaccine stimulations, with post hoc Tukey’s multiple comparison analysis. Statistical significance was only observed in the cells stimulated with IPR8-GMBS-R848 at 10 μg/mL compared to the other stimulations (excluding the TLR mix). * *p* = < 0.05, ** *p* = < 0.005, *** *p* = < 0.0005.

Human moDCs are a tractable model for gaining a mechanistic understanding of how APCs are differentiated in vitro. However, it is still unclear whether they deviate in response compared to cells differentiated in vivo. Thus, we sought to extend our finding that the GMBS-conjugated vaccine was more stimulatory to cells that were differentiated in vivo. This analysis also allowed us to examine other cell types beyond DCs. PBMCs from healthy donors were stimulated ex vivo for 24 h with IPR8, IPR8-SM(PEG)_4_-R848, IPR8-GMBS-R848 (1 µg/mL or 10 µg/mL), or were left non-stimulated (NS). TNFα (Figure 5A) and IL-12p70 (Figure 5B) production significantly increased in cells stimulated with 10 µg/mL of the IPR8-GMBS-R848 vaccine. Although not reaching statistical significance, 1 µg/mL of IPR8-GMBS-R848 vaccine also resulted in increases in the cellular production of proinflammatory cytokines. Interestingly, in contrast to TNFα and IL-12p70, we observed no statistically significant difference in IFNα production in the vaccine-stimulated cells compared to the non-stimulated cells (Figure 5C). These data suggest that stimulation with the GMBS-linked vaccine promotes greater production of proinflammatory cytokines ex vivo in human PBMCs.

Next, we sought to identify cell populations within the PBMCs that were responding to the vaccine. APC were identified via positive staining for CD11c and HLA-DR along with negative staining for CD3 and CD20 (T and B cell markers) (Figure 5D).

Stimulation with IPR8-GMBS-R848 for 16 h significantly increased the percentage of TNFα producing HLA-DR+ CD11c+ cells (Figure 5E,F). We also assessed the percent of IL-12p70 cells in PBMCs stimulated with the different vaccines after 16–20 h; however, the response was not readily detectable, even with soluble R848 (data not shown). The significant, but low amount of IL-12p70 detected in bulk PBMC cultures (Figure 5) together with the small percentage of IL−12p70+ moDCs (Figure 1D, left panel) lead us to conclude that IL-12p70-producing cells are below the limit of detection via flow cytometry.

**Figure 6:**
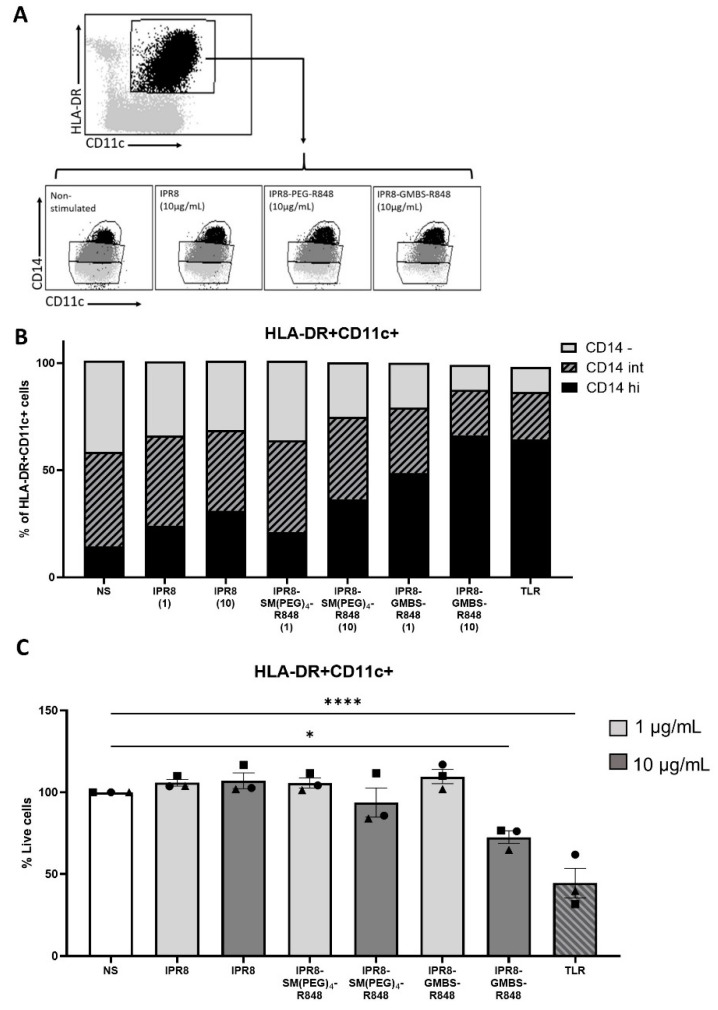
Increasing the stimulatory capacity of the inactivated influenza virus vaccines results in a concomitant increase in the proportion of APCs that express CD14. PBMCs were isolated from healthy adult donors and stimulated with IPR8, IPR8-SM(PEG)_4_-R848, IPR8-GMBS-R848 (1 μg/mL and 10 μg/mL), a TLR cocktail as a positive control (MPLA 1 μg/mL, PolyI:C 1 μg/mL, and R848 10 μM), or were left non-stimulated (NS) for 16 h. (**A**) Gating strategy of CD14+ APCs. (**B**) The percentage of HLA-DR + CD11c+ cells that are CD14 high (hi) CD14 Intermediate (int) or CD14 negative (-). (**C**) Quantification of the percentage of HLA-DR + CD11c+ cells within the live cell populations. Data represent mean ± SEM from 3 donors. Symbols on graph represent individual donors. Statistical significance was assessed using a one-way ANOVA with post hoc Tukey’s multiple comparison analysis. * *p* = < 0.05, **** *p* = < 0.0001.

In our flow cytometric analysis, we observed that the TNFα-producing cells had a high expression of the surface marker CD14. We were curious to understand the relationship between the expression of CD14 within the HLA-DR+ CD11c+ cells and TNFα production. CD14 is a general marker that can delineate monocytes from DCs, as CD14 expression through human peripheral DCs has not been extensively reported. In our analysis, we observed an unexpected and profound shift in the expression of CD14 within the HLA-DR+ CD11c+ population that was correlated with the stimulatory activity of the vaccines (Figure 6A,B). As demonstrated in Figure 6B, not only did CD14 expression increase with increasing stimulatory capacity (i.e., GMBS > SM(PEG)4 > IPR8), but the shift was also apparent when increasing the dose of an individual vaccine, i.e., from 1 µg/mL to 10 µg/mL.

Although we did observe moderate levels of cell death in the high dose IPR8-GMBS-R848 and TLR cocktail stimulated groups, the shift in CD14 expression cannot be solely attributed to this, as an increase in the proportion of CD14 expressing APCs under conditions that do not increase cell death was observed in the IPR8-GMBS-R848 1 µg/mL stimulated cells (Figure 6C). These data support a model wherein stimulation is driving increased expression of CD14 on HLA-DR+ CD11c+ cells.

### 3.7. The GMBS-Conjugated Vaccine Promotes Increased PR8-Specific IgG Antibody Compared to the SM(PEG)_4_-Conjugated and IPR8 Vaccines

Based on our finding of increased stimulatory activity of the IPR8-GMBS-R848 vaccine when added to cells cultured in vitro and ex vivo, we assessed whether it would exhibit increased activity in vivo. We reasoned this was possible given studies showing that cytokines from APCs influence the activation and differentiation of T helper cells that direct the magnitude and nature of the humoral immune response [42].

**Figure 7:**
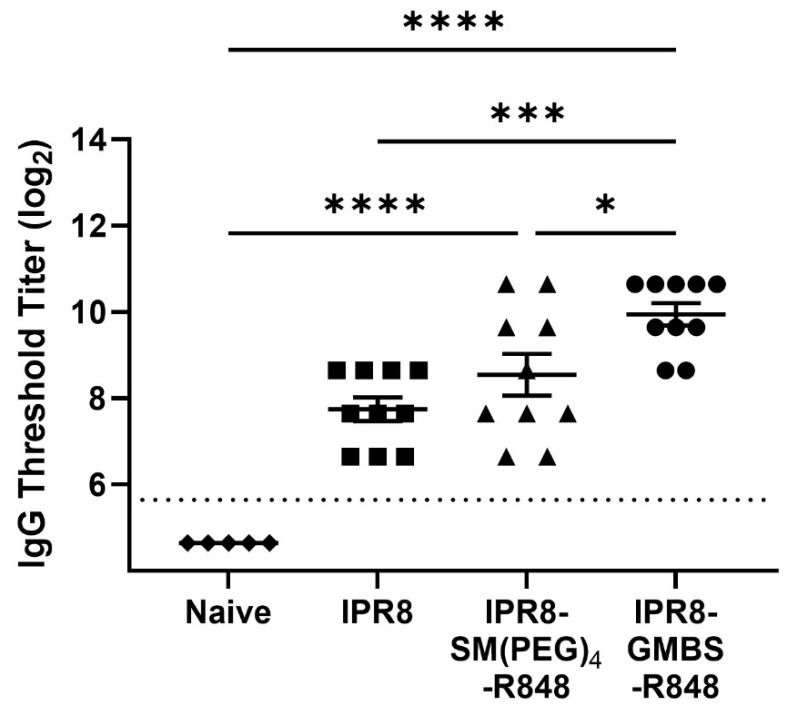
Vaccination with IPR8-GMBS-R848 results in increased PR8-specific IgG antibody. BALB/c mice (n = 10) were vaccinated intramuscularly (i.m.) with 0.03 µg of IPR8, IPR8-SM(PEG)_4_-R848, or IPR8-GMBS-R848. Blood was collected 14 days p.v. Naïve BALB/c mice (n = 5) serum was used as a negative control. The circulating levels of PR8-specific total IgG was assessed by ELISA. Threshold titer (TT) was defined as the highest dilution at which the sample’s optical density was at least 3 times that of the assay background. The limit of detection is represented by the dashed line. Data represent mean ± SEM from mice vaccinated in two independent experiments. Symbols on graph represent different vaccine groups. Statistical significance was assessed using a one-way ANOVA with post hoc Tukey’s multiple comparison analysis * *p* = < 0.05, *** *p* = < 0.0005, **** *p* = < 0.0001.

Influenza places a significant burden on infant health globally. Influenza vaccines are administered to humans starting at 6 months of age. As a result, depending on the timing of birth, humans likely receive their first dose at 6–12 months of age. This is also the time when humans are most likely to be naive. Thus, we chose to probe the potential of these vaccines in the context of an infant/young child mouse model (3 week old BALB/c mice). BALB/c mice were injected (i.m.) with 0.03 µg of IPR8, IPR8-SM(PEG)_4_-R848, or IPR8-GMBS-R848 vaccine. PR8-specific IgG antibody in the serum was assessed at 14 days p.v. We observed a statistically significant increase in the circulating PR8-specific total IgG in mice vaccinated with the GMBS-linked vaccine compared to mice vaccinated with the SM(PEG)_4_-linked vaccine or IPR8 alone (Figure 7). These data show that R848 conjugation using the GMBS linker resulted in an inactivated influenza vaccine with increased stimulatory activity in vivo.

## 4. Discussion

While vaccines have been a tremendous success story, it is clear that there is a need to increase the effectiveness of current approaches in order to generate optimal immune responses, especially in those with less responsive immune systems. This has led to a significant focus on identifying adjuvants that can increase the stimulatory capacity of vaccines. In our previous studies, we discovered that linkage of the TLR7/8 agonist R848 to the influenza virion resulted in potent immunostimulatory activity greater than that of virion alone, both in vitro and in vivo [7,8,9,10]. Subsequently, we found the linker used for conjugation impacts the ability to stimulate in vitro differentiated DCs [33]. Here, we extend our findings showing that the increase with the GMBS-conjugated vaccine was the combined result of an increased percentage of moDCs that produced cytokine and more cytokine production per cell. These findings were supported with results from bona fide APCs differentiated in vivo. The increased stimulatory activity was associated with increased activation of the NF-κB pathway. Critically, we show that the increased stimulatory activity translates to improved antibody responses following vaccination.

We were intrigued with the question of how the IPR8-GMBS-R848 vaccine produced this effect. We had previously found that the association of the vaccine with moDCs was similar [33]. Here, we show that it is not the result of increased R848 conjugation or aggregation. The latter was of interest as prior studies evaluating the impact of size on dendritic cell uptake capacity found that that virus-sized particles of ~20–200 nm are optimal [43,44,45]. Further, an elegant study by Seder and colleagues showed the size of TLR7/8-agonist-conjugated synthetic polymers profoundly impacted uptake by and maturation of DCs and monocytes [27].

Although we observed no appreciable changes in cell association, R848 conjugation, or size, we did find significant differences in TLR signaling with the GMBS- versus the SM(PEG)_4_-conjugated vaccines. The GMBS vaccine promoted increased NF-κB activity as shown through the increased phosphorylation of IκBα. We initially hypothesized that changes in TLR8 signaling could be qualitative or quantitative. The increase in NF-κB activity and increased production of TNFα and IL-12p70 in GMBS-vaccine-stimulated cells would suggest a quantitative effect of the GMBS versus SM(PEG)_4_ vaccine constructs is, at least, partially responsible. How might this be occurring? TLR7 and TLR8 reside in the endosome as homodimers [46] and require proteolytic cleavage before engagement with ssRNA [46,47,48]. We expect that R848 is released as a result of hydrolases in the endosomal compartment. The release of R848 may be differentially sensitive to this process as a result of the linker used, thus, potentially altering the effectiveness of TLR engagement. An alternative hypothesis for the changes in TLR signaling could be, in part, a result of increased vaccine internalization and or trafficking to the endosome. Further study is warranted to investigate these possibilities.

An interesting finding from our PBMC studies was the upregulation of CD14 on CD11c+ cells following stimulation. The percent of cells expressing CD14 was strongly correlated with the degree of stimulation in the cultures as read out by cytokine production. Previous studies have reported upregulation of this molecule on dendritic cells in the presence of IL-1β [49]. The increased expression in our studies is in keeping with our previous analyses of moDCs, where we found much higher levels of IL-1β produced following stimulation with IPR8-GMBS-R848 compared to IPR8-SM(PEG)_4_-R848 [33]. While DCs are well accepted as a critical cell type for activation of naïve T cell responses, there are data supporting a role for monocytes via antigen presentation and through cytokine production [50,51,52]. In a humanized mouse model, CD14^+^ DCs were found to polarize CD4^+^ T cells along the TH1 differentiation pathway [53]. The production of IL-12p70 by IPR8-GMBS-R848 would suggest activation of APC capable of driving TH1 skewing.

Our previous work showed improved immunogenicity in newborn AGM vaccinated with IPR8-SM(PEG)_4_-R848 vaccine compared to those vaccinated with IPR8 with non-adjuvant [9]. Interestingly, we did not observe increases in costimulatory molecules on newborn DCs from the draining LN analyzed 24 h after vaccination with the IPR8-SM(PEG)_4_-R848 compared to animals vaccinated with IPR8 in the absence of adjuvant [8]. Our recent finding that the GMBS crosslinker is more effective at inducing APC maturation and proinflammatory cytokine production in RAW264.7 cells [33], human moDCs [33], and PBMCs suggests this construct may have improved activity in vivo. We found this to be true, showing that the GMBS-linked vaccine resulted in higher influenza-virus-specific IgG compared to IPR8-SM(PEG)_4_-linked or IPR8 vaccine in vaccinated mice.

It is well-established that newborn DCs have a reduced ability to upregulate the expression of costimulatory molecules and to produce IL-12p70 as a result of diminished TLR responsiveness to PAMPs [54,55,56,57]. Our findings suggest that the GMBS crosslinker may have the capacity to improve the efficacy of an IPR8-R848 vaccine in infants through improved maturation and proinflammatory cytokine production in APC. Further studies are required to address this possibility.

The need for improved vaccines has spurred much research aimed at the discovery of new adjuvants as well as the optimal delivery of those already known to possess stimulatory activity. Here, we provide new insights into the improved function conferred by the nature of the linkage of the TLR7/8 agonist R848 to a viral particle. The demonstration that the GMBS-linked vaccine results in a more potent response in vivo supports further investigation of this approach.

## Figures and Tables

**Table 1:** Characteristics of the different vaccine constructs.

Vaccine	R848/mL for 100 µg Protein	Z-Average	PDI
IPR8	-	142 d.nm	0.099
IPR8-SM(PEG)_4_-R848	8.12 nmoL/mL	138.3 d.nm	0.137
IPR8-GMBS-R848	6.05 nmoL/mL	138.6 d.nm	0.129

## Data Availability

The datasets generated for this study can be found upon request to the corresponding author.
